# Patterns of genetic variation in populations of infectious agents

**DOI:** 10.1186/1471-2148-7-116

**Published:** 2007-07-13

**Authors:** Isabel Gordo, Paulo RA Campos

**Affiliations:** 1 Instituto Gulbenkian de Ciência, P-2781-901 Oeiras, Portugal; 2 Departamento de Física, Universidade Federal Rural de Pernambuco 52171-900, Dois Irmãos, Recife-PE, Brazil

## Abstract

**Background:**

The analysis of genetic variation in populations of infectious agents may help us understand their epidemiology and evolution. Here we study a model for assessing the levels and patterns of genetic diversity in populations of infectious agents. The population is structured into many small subpopulations, which correspond to their hosts, that are connected according to a specific type of contact network. We considered different types of networks, including fully connected networks and scale free networks, which have been considered as a model that captures some properties of real contact networks. Infectious agents transmit between hosts, through migration, where they grow and mutate until elimination by the host immune system.

**Results:**

We show how our model is closely related to the classical SIS model in epidemiology and find that: depending on the relation between the rate at which infectious agents are eliminated by the immune system and the within host effective population size, genetic diversity increases with *R*_0 _or peaks at intermediate *R*_0 _levels; patterns of genetic diversity in this model are in general similar to those expected under the standard neutral model, but in a scale free network and for low values of *R*_0 _a distortion in the neutral mutation frequency spectrum can be observed; highly connected hosts (hubs in the network) show patterns of diversity different from poorly connected individuals, namely higher levels of genetic variation, lower levels of genetic differentiation and larger values of Tajima's D.

**Conclusion:**

We have found that levels of genetic variability in the population of infectious agents can be predicted by simple analytical approximations, and exhibit two distinct scenarios which are met according to the relation between the rate of drift and the rate at which infectious agents are eliminated. In one scenario the diversity is an increasing function of the level of transmission and in a second scenario it is peaked around intermediate levels of transmission. This is independent of the type of host contact structure. Furthermore for low values of *R*_0_, very heterogeneous host contact structures lead to lower levels of diversity.

## Background

Patterns of genetic diversity in populations of infectious agents contain important information about their epidemiology and evolution. They depend on the population dynamics of the infectious agents, which involves their replication within hosts and transmission between hosts, their mutation and recombination rate. Infectious agents vary enormously in their ability to mutate and to transmit, which will lead to large differences in levels of variability. Furthermore there can be variation within an infectious species for the ability to evade the host immune system. In fact, infectious agent genetic diversity can help in targeting genes under selection pressure created by the immune system [1]. In addition patterns of infectious agent variation can, under certain circumstances, be used to infer host population history [2], and the level of infectious agent genetic structure may reflect its evolutionary potential [3]. Importantly, the need for a continuous integration between population genetics and epidemiology has been increasingly recognized [4-7].

In population genetics the standard neutral model has a long history in DNA sequence data analysis [8], and has been extensively used as a null model for understanding genetic variation in natural populations, including that in our own species [8,9]. The standard neutral model makes several simplifying assumptions: in particular it makes the simple assumption that individuals form one single constant size population. When considering populations of infectious agents it is much more reasonable to assume, as the null model, a population composed of a collection of much smaller populations.

Here we develop population genetics models of structured populations, that incorporate epidemiological parameters explicitly, in order to study genetic variability under one of the simplest possible epidemiological models. We ask mainly two questions: 1) what do levels and patterns of sequence variation in these infectious agents look like under this model? And 2) how does host contact structure influence their diversity?

The models we will study here are very similar to the metapopulation models where each subpopulation can go extinct and be recolonized [10-12]. Generally studies of genetic diversity in such subdivided populations [13,14] assume a simple symmetric topology for the metapopulation – the most well studied is the island model of Wright. Simple as it is, this model has provided a wealth of results that have led to enormous contributions to our understanding of evolution in structured populations [15,16]. Nevertheless, there are several reasons to think that this model is too simple to be readily applicable to natural populations [14,17], especially if the goal is to understand molecular diversity of infectious agents. As we know, the underlying topology at which certain disease epidemics and spreading takes place is that of social networks [18]. Several recent investigations have demonstrated that real networks of interaction have a much more complex structure than those predicted by totally regular networks or totally random networks [19]. Most real networks of social interactions present two different topological properties: a low average pairwise distance between nodes and a high clustering degree (which measures local structuring).

The former occurs in random networks and the latter in regular networks. In such way, some models of network topologies have been recently proposed in the literature (for a review see Ref. [20]). One of the most successful models for network structure is the scale-free network [21]. In addition to the common properties of real interaction networks, in scale-free networks the distribution of connectivities obeys a power-law distribution as P(ki)∝ki−γ
 MathType@MTEF@5@5@+=feaafiart1ev1aaatCvAUfKttLearuWrP9MDH5MBPbIqV92AaeXatLxBI9gBaebbnrfifHhDYfgasaacH8akY=wiFfYdH8Gipec8Eeeu0xXdbba9frFj0=OqFfea0dXdd9vqai=hGuQ8kuc9pgc9s8qqaq=dirpe0xb9q8qiLsFr0=vr0=vr0dc8meaabaqaciaacaGaaeqabaqabeGadaaakeaacqWGqbaucqGGOaakcqWGRbWAdaWgaaWcbaGaemyAaKgabeaakiabcMcaPiabg2Hi1kabdUgaRnaaDaaaleaacqWGPbqAaeaacqGHsisliiGacqWFZoWzaaaaaa@3979@, which is observed in some actual systems ranging from World Wide Web to the network of human sexual contacts [22,23]. As initially proposed, scale-free networks are dynamical networks where growth and preferential attachment are some of the key mechanisms.

Accordingly, each newly introduced node in the network preferentially joins with an already well connected-node. As a result, it will produce a highly heterogeneous network where most nodes have a low connectivity while a few nodes display a very large connectivity. These latter ones are referred to as hubs. The understanding of the interplay between the underlying topology and the forces driving systems is of crucial relevance [24,25]. One example of this, that has received a great deal of attention, is that of network epidemiology: the study of epidemic and disease spreading [26-29], which are strictly tied to the topology of social contact networks. In this context, a striking result has arisen from the study of the classical susceptible-infected-susceptible (SIS) epidemiological model on scale free networks: scale-free networks are more prone to spreading of diseases than random graphs and regular lattices [26,27]. In this kind of model the role of microbe evolution is disregarded. Recently, we have focused on this latter feature and we have shown that although scale-free networks are more prone to infectious agent spread, the accumulation of deleterious mutations in asexual infectious agent with high mutation rates can also be accelerated in this kind of networks in comparison to random graphs [30]. This shows that not only disease dynamics but also its evolution should be considered as an important key in the investigation of epidemiological models [7]. Another very important feature that has to be considered is co-evolution between infectious agent and their hosts [31]. Modeling of these complex systems have provided us with insights into how host-parasite interactions can modulate the mode of reproduction [32], ploidy levels [33], the patterns of gene expression in hosts and parasites [34] and how different types of interspecies interactions affect genetic and phenotypic variation [35].

## Results and Discussion

### Levels of metapopulation infection

The susceptible-infected-susceptible model (SIS model) is one of the simplest classical models in epidemiology. In this model, hosts born susceptible (S) can become infected (I) at a rate *β *per unit time, given contact with at least one infected host. Infected hosts become susceptible at a rate *λ*, such that 1/*λ *is the average duration of an infection. One of the most fundamental quantities to assess the equilibrium frequency of infections in the population is the *R*_0 _of the infectious agent. The *R*_0 _is defined as the number of secondary cases produced by an infectious individual in a totally susceptible population. At epidemiological equilibrium, the frequency of infected individuals is *i *= 1 - 1/*R*_0_, with *R*_0 _= *β*/*λ*. If *R*_0 _< 1 then the infection does not spread.

To assess the patterns of variation under the SIS model, we have studied a population genetic model of a structured population that is composed of many small subpopulations, which are named demes. There is a total of *D *demes, which are connected according to a given network topology: corresponding to either the island model or the scale free network. These demes can go extinct and be recolonized through migrants that they received from the other demes. Each deme can contain at most *N*_*d *_individuals, which reproduce and mutate within each deme (see Methods). In Table 1 we make a summary of the model's key parameters.

**Table 1:** Model parameterization

parameter	meaning in metapopulation genetics	meaning in epidemiology
*D*	number of demes	number of hosts
*N*_*d*_	number of individuals within a deme	number of infectious agents within an infected host
*e*	probability that a deme goes extinct	probability that the immune system clears the infection
*m*	migration rate	transmission ability between hosts
*μ*	mutation rate	mutation rate of the infectious agent
*k*_*j*_	number of demes connected to deme *j*	number of contacts of host *j*

We now relate our metapopulation model with the SIS model and in this study we will ask what equilibrium patterns of infectious agent genetic variation look like under this model. In our model a deme corresponds to a host. An empty deme means that the host is susceptible, whereas a deme which is full corresponds to an infected host. A deme that is currently full can become empty with probability *e*, which means that *e *corresponds to *λ*. A deme that is currently empty can become full through the migrants it receives from nearby demes. This implies that *β *is proportional to *m*. Given that the average connectivity of a deme is *K *and that the number of migrants per link is *N*_*d*_*m*, then *β *corresponds to *N*_*d*_*mK*.

In order to assess the correspondence between our model and the SIS model, we have compared the average frequency of infected individuals in our metapopulation with the expectation for the deterministic SIS model, which implies that:

*i *= 1 - 1/*R*_0 _= 1 - *e*/*N*_*d*_*mK*

Equation 1 is the expected frequency when there is no variance in *k*_*i*_, which is not the case in scale free networks.

**Figure 1:**
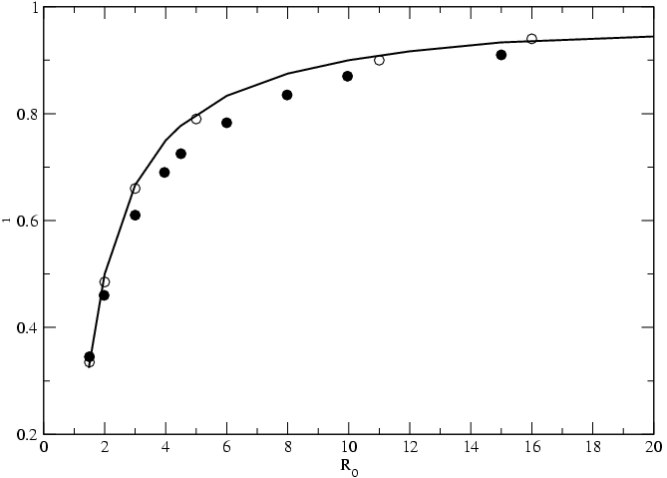
**Infected Individuals**. The proportion of infected individuals, *i*, with increasing *R*_0 _= *N*_*d*_*mK*/*e*. Full circles are the results for scale free networks (with *γ *= 3) and empty circles for the island model. *D *= 900, *N*_*d *_= 10, *e *= 0.01 in all network topologies. The line denotes the expected value of *i *under the deterministic SIS model.

In Figure 1 we show the results from our simulations, where the proportion of infected individuals in the metapopulation is measured as we increase the transmission coefficient of the infectious agent, *β*, through increments in *m*. The results for the different types of networks considered are shown, and the deterministic expectation is also plotted. In all cases *R*_0 _= *N*_*d*_*mK*/*e*, where *K *= *D *- 1 for the island model and *K *= 6 for the scale free topology. The results of the simulations show that the proportion of infected individuals observed and that predicted are quite concordant. In particular, if we assume the topology corresponding to the island model, then the level of infection is exactly that predicted by Equation 1. We notice that the prediction holds for an effectively infinite population under the mass action assumption.

One may expect deviations to be observed when these assumptions are violated [36]. Nevertheless the deviations we observe are small, unless *R*_0 _is very low. In fact in the case of very low *R*_0 _there is a high probability that the infection does not spread. For example in the scale free network, if the infection starts in a poorly connected host it may have very little chance of spreading. We performed simulations with the scale free topology in conditions where the infection starts in a single randomly chosen host. With the same parameters as in Figure 1 and for *R*_0 _= 1.5, we observed 66% of cases where the disease could not spread. With *R*_0 _= 3, the fraction of cases where the infectious agent could not invade dropped to 40%.

### Levels of metapopulation diversity

**Figure 2:**
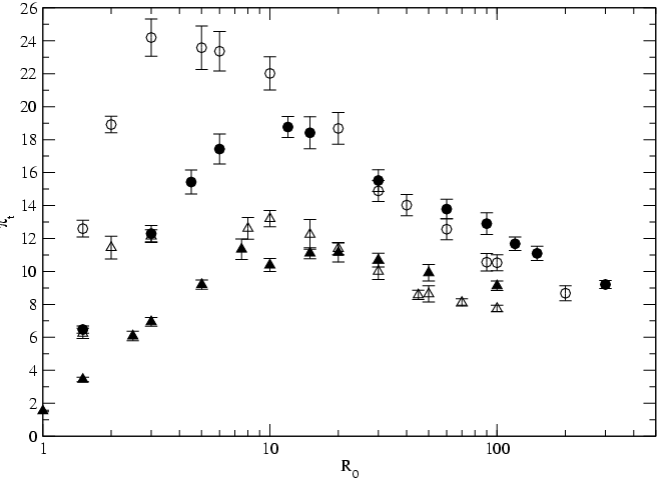
**Diversity in the metapopulation**. The level of diversity *π*_*t *_as a function of *R*_0 _= *N*_*d*_*mK*/*e*. The parameters values are *D *= 1000, *N*_*d *_= 10, *n*_*t *_= 50 and *μ *= 0.0004. The empty symbols denote the results for the island model while the full symbols correspond to scale-free networks with *γ *= 3. The results for *e *= 0.01 are represented by circles and *e *= 0.02 by triangles.

We now study the level of genetic diversity in infectious agents sampled randomly from the whole population of infected hosts. We first consider a metapopulation where every host contacts every other host. This corresponds to the island model in the populations genetics literature and mass action assumption in epidemiological models. We then assess how the level of diversity is affected by differences in the level of contact between hosts, in particular when a small number of hosts can have a very large number of contacts, such as in the scale free network. In Figure 2 we show the level of diversity in samples taken from the whole population, *π*_*t*_, as we increase *R*_0_, through increments in *m*.

We observe that, for both topologies and for the sets of parameters considered, the level of *π*_*t *_is maximal for intermediate values of *R*_0_. For instance, when *e *= 0.01 this maximum value is achieved at *R*_0 _around 3 for the island model and around 10 for the scale free topology. Beyond these points the level of diversity starts to decrease with increasing *R*_0_. From Figure 2 we observe the occurrence of two quite distinct regimes, according to the level of transmission. In the region of low transmission, *R*_0 _is small, extinction is much stronger than migration (*e *>> *mK*), the fraction of infected hosts is small and levels of diversity are low. In fact, starting from *R*_0 _= 1, where the fraction of infected individuals, *i*, is 0, as we increase *R*_0 _(by increasing *m*), the level of infection rises and the level of diversity accompanies that increase. In this region the level of infection bounds the level of diversity in the population, since it is expected that diversity will be higher when the total number of infectious agents in the metapopulation is larger. When the level of infection achieves a value close to 0.9, increments in *m*, lead to small increments in *i *and the level of diversity stops increasing. The second regime comes about at high transmission, where *R*_0 _is very large. In this region migration is much stronger than extinction, *mK *>> *e*, the level of infection is close to 1 and so it is not the limiting factor for diversity to grow. From this point, increments in migration cause a drastic reduction in the isolation between demes and lead to a reduction in diversity. In fact in the limit of extremely high levels of migration the diversity in the structured population tends to that expected in a panmitic population of size *N*_*t *_= *DN*_*d*_. So, for very high values of *R*_0_, diversity tends toward the value *π*_*t *_= *π*_*d *_= 2*N*_*d*_*D*_*μ*_, which in the case of Figure 2 is 8, for the value of the mutation rate, *μ*, assumed. Figure 2 also shows that in the region of low *R*_0_, diversity in the island model is higher than in the scale free network, whereas for large values of *R*_0_, there is little difference between the topologies. The latter is expected since the larger the value of the migration rate the less important the precise contact structure will be. The former can be understood as follows: a low value of *R*_0 _corresponds to a small fraction of infected hosts both in the island model and in the scale free network. But whereas in the island model new infections of a susceptible host occur from contact with any of the infected hosts in the metapopulation, in the scale free network infections are more likely to come from well connected hosts, which are a small subset of the metapopulation. This then will lead to lower diversity levels in the scale free network, as compared to the island model, for the same low *R*_0 _value.

We have compared our simulation results with some of the analytical approximations for the levels of diversity in metapopulations [13]. In the vast majority of metatopulation models with extinction and recolonization, the island model of population subdivision is assumed. Furthermore, the processes of migration and recolonization are assumed to be distinct. Two different schemes of colonization are normally considered, according to where colonists come from: the migrant pool model and the propagule pool model [14,37,38]. In both models there are *k *colonists (where *k *is a fixed number independent of migration), which may constitute a random sample from the whole metapopulation (migrant pool model) or from a single deme (propagule model). Pannell and Charlesworth [13] have studied levels of within and between population diversity under these models and have provided a set of analytical approximations. We have adapted the approximations in their Table 2, which correspond to the infinite sites mutation model as we assume here, to the metapopulation model that we are studying, which is slightly different from the one they have used. In particular, besides the different types of contact structure studied here, there are two key differences in the models: 1) in our model recolonization and migration are similar processes; and 2) while in the classical model it is assumed that when one deme goes extinct it gets immediately recolonized, in our model when a deme goes extinct it will only be recolonized when it receives migrants. In this way, the equilibrium number of empty demes (susceptible individuals) decreases as *m*, or *R*_0_, increases. Whereas for infectious agent populations assumption 2) is more appropriate, for some infectious agents assumption 1) may be too simple. One can imagine that when a host is infected, its ability to transmit the infectious agents to another infected host is reduced compared to its ability to transmit the infectious agent to a susceptible individual. This implies that the migration rate between subpopulations may, in some infectious agents, depend on the host history. We have taken the simplest scenario here.

**Table 2:** Mean values of Tajima's D in the scale free network with parameters

*n*_*t*_	*D*_*t *_2*SE*
10	-0.103 0.095
25	-0.190 0.076
75	-0.328 0.110
150	-0.274 0.114
250	-0.392 0.110
300	-0.422 0.092

Parameter values: *D *= 1000; *N*_*d *_= 10; *μ *= 0.0004, *e *= 0.01, *R*_0 _= 1.5

We have thus compared the expected level of metapopulation genetic diversity in our simulations for the symmetric island model (where every host contacts every other host) with the following approximation:

πtisl=2NdD(1−1/R0)μR0+1/2eNd+R0
 MathType@MTEF@5@5@+=feaafiart1ev1aaatCvAUfKttLearuWrP9MDH5MBPbIqV92AaeXatLxBI9gBaebbnrfifHhDYfgasaacH8akY=wiFfYdH8Gipec8Eeeu0xXdbba9frFj0=OqFfea0dXdd9vqai=hGuQ8kuc9pgc9s8qqaq=dirpe0xb9q8qiLsFr0=vr0=vr0dc8meaabaqaciaacaGaaeqabaqabeGadaaakeaaiiGacqWFapaCdaWgaaWcbaGaemiDaq3aaSbaaWqaaiabdMgaPjabdohaZjabdYgaSbqabaaaleqaaOGaeyypa0JaeGOmaiJaemOta40aaSbaaSqaaiabdsgaKbqabaGccqWGebarcqGGOaakcqaIXaqmcqGHsislcqaIXaqmcqGGVaWlcqWGsbGudaWgaaWcbaGaeGimaadabeaakiabcMcaPiab=X7aTnaalaaabaGaemOuai1aaSbaaSqaaiabicdaWaqabaGccqGHRaWkcqaIXaqmcqGGVaWlcqaIYaGmcqWGLbqzaeaacqWGobGtdaWgaaWcbaGaemizaqgabeaakiabgUcaRiabdkfasnaaBaaaleaacqaIWaamaeqaaaaaaaa@50C9@

which is adapted from the approximation for the classical case of the migrant pool model of recolonization. We can expect that in the case of the scale free topology, where a large number of hosts have few connections and a few hosts are very well connected, levels of diversity will be closer to those expected for the propagule pool recolonization model. This is because hubs in the network will contribute much more than the other nodes in the process of recolonization. We have thus compared the expected level of genetic diversity for the scale free network with the following approximation for the propagule pool model:

πtsf=D(1−1/R0)μ1−ee(2−e)
 MathType@MTEF@5@5@+=feaafiart1ev1aaatCvAUfKttLearuWrP9MDH5MBPbIqV92AaeXatLxBI9gBaebbnrfifHhDYfgasaacH8akY=wiFfYdH8Gipec8Eeeu0xXdbba9frFj0=OqFfea0dXdd9vqai=hGuQ8kuc9pgc9s8qqaq=dirpe0xb9q8qiLsFr0=vr0=vr0dc8meaabaqaciaacaGaaeqabaqabeGadaaakeaaiiGacqWFapaCdaWgaaWcbaGaemiDaq3aaSbaaWqaaiabdohaZjabdAgaMbqabaaaleqaaOGaeyypa0JaemiraqKaeiikaGIaeGymaeJaeyOeI0IaeGymaeJaei4la8IaemOuai1aaSbaaSqaaiabicdaWaqabaGccqGGPaqkcqWF8oqBdaWcaaqaaiabigdaXiabgkHiTiabdwgaLbqaaiabdwgaLjabcIcaOiabikdaYiabgkHiTiabdwgaLjabcMcaPaaaaaa@4808@

which is valid only when *mK *<*e *[13]. We therefore expect this expression to provide a good approximation for cases in which *R*_0 _<*N*_*d*_.

**Figure 3:**
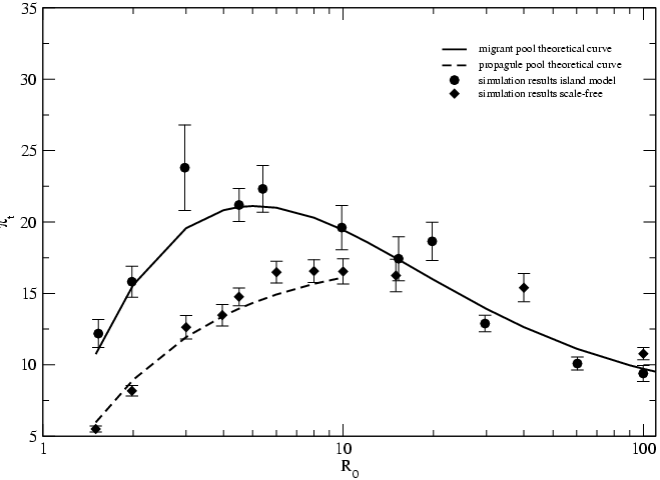
**Theoretical approximations and the different topologies**. Comparison of the level of diversity *π*_*t *_between topologies and with the theoretical approximations. *D *= 900, *N*_*d *_= 10, *e *= 0.01, *n*_*t *_= 50 and *μ *= 0.0004 in all networks.

As seen in Figure 3, these formulas provide very good approximations to the simulation results, for low values of *R*_0_. For very large values of *R*_0_, the level of diversity is similar in the two topologies and is very well approximated by Equation 2.

Equation 2 suggests a strong dependence of the level of metapopulation diversity with *N*_*d*_, the effective population size within a host. This effective population size is likely to vary considerably among different infectious agent species. We have therefore explored how the value of *N*_*d *_affects the levels of diversity with simulations.

**Figure 4:**
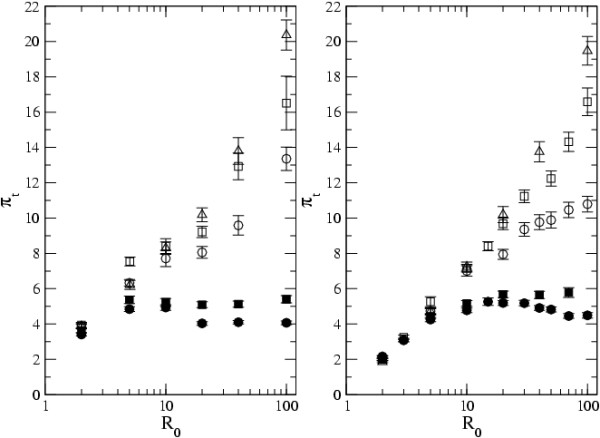
**Effect of *N*_*d *_in metapopulation diversity**. The level of diversity, *π*_*t*_, with *R*_0 _and *N*_*d *_in the island model (left panel) and scale free networks (right panel). *N*_*d *_= 5 in full circles, *N*_*d *_= 7 in full squares, *N*_*d *_= 20 in open circles, *N*_*d *_= 40 in open squares and *N*_*d *_= 60 in open triangles. Other parameters are *D *= 1000, *e *= 0.05, *n*_*t *_= 50 and *μ *= 0.0004 for all networks.

In Figure 4 we show the results of varying *N*_*d*_, for both types of network (island model in the left panel and scale free network in the right panel). Figure 4 clearly shows that when *e *< 1/*N*_*d *_(filled symbols in both panels), levels of diversity are maximal for intermediate *R*_0_. But for *e *> 1/*N*_*d *_diversity always increases with *R*_0_. This occurs both in the island model and in scale free networks. This shows that, independently of the type of host contact structure, for infectious agents with large intrahost effective population size, levels of diversity increase with increasing *R*_0_.

Furthermore, as suggested by Equation 2, for small values of *R*_0_, increasing *N*_*d *_has a very small effect on the level of diversity, but for intermediate to high *R*_0 _values the effect is more pronounced.

When *R*_0 _> 10, the level of infection is not a limiting factor in the level of diversity, because the number of infected hosts is very high. Thus for large values of *R*_0 _infectious agent diversity will increase with *N*_*d*_.

Comparing the panels in Figure 4, we can observe that when *R*_0 _<< 10, diversity is always smaller in the scale free network, whereas when *R*_0 _>> 10 and *e *> 1/*N*_*d *_the levels of diversity are similar in both contact networks. In fact, for large values of *R*_0_, the largest difference between the topologies can be observed when *e *= 1/*N*_*d*_.

In this metapopulation model there are two forces which generate diversity within each host: mutation and transmission; there are also two forces that undermine diversity: extinction and genetic drift. So in general, we can expect that, when the forces that reduce diversity are stronger than those that generate it (that is low *R*_0_, low *N*_*d *_or high *e*), diversity levels will be low. On the contrary, high *R*_0_, high *N*_*d *_or low *e*, we can expect levels of diversity to be much larger.

### Metapopulation mutation frequency spectrum

The spectrum of frequencies of mutations that are segregating in the population is important to understand deviations from the standard neutral model, which assumes an undivided, constant size population at equilibrium between mutation and drift [39]. In fact, the mutational spectrum of infectious agent gene sequences has been used to reject the standard neutral model suggesting that natural selection is determining the evolution of certain genes [40,41]. Tajima's D is a widely used statistic to assess distortions in the frequency spectrum [42]. If the number of mutations that appear at frequency 1/*n *in sample of size *n *(singletons) is higher than that expected under the standard neutral model, then Tajima's D becomes negative. On the other hand if the number of mutations at intermediate frequency is large then Tajima's D becomes positive. When a departure from the standard neutral model is observed in a given gene of a given species, several alternative hypotheses can be made. These typically involve natural selection and/or demographic factors, such as population growth or population structure. In infectious agent populations the relevant null model against which we would like to test for the molecular signature of selection is closer to a metapopulation neutral model than to the standard neutral model. From all the simulations in all the metapopulation structures we have studied, we have observed that *D*_*t *_was always very close to 0. This is in agreement with the results of coalescent theory and simulations in metapopulations under the island model [43,44]. However, we have observed that in some simulations of scale free networks a slight distortion of the frequency spectrum was apparent. In cases of low *R*_0 _mean values of the Tajima's D statistic become negative. In Table 2 we show one example where this occurs. Although the values of *D*_*t *_are not very negative when the sample size is small, they become more negative with increasing sample size.

**Figure 5:**
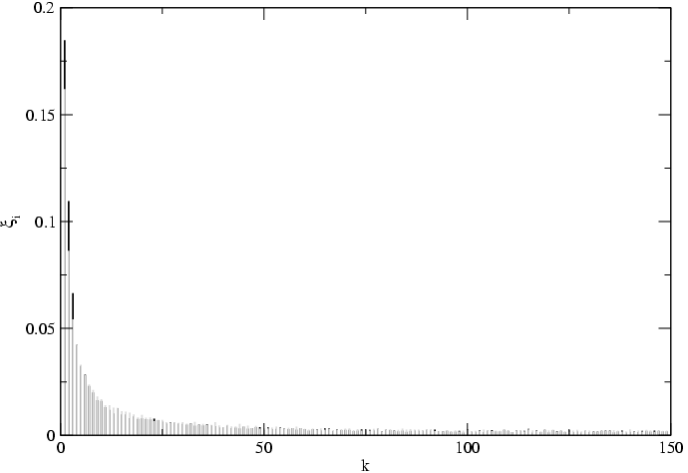
**Frequency spectrum**. The frequency spectrum of neutral mutations in scale free networks with *γ *= 3. In the Y-axis we plot the probability that in a sample of size *n*_*t *_= 300 we find mutations with frequency *k*/*n*_*t *_or with frequency (*n*_*t *_- *k*)/*n*_*t*_. *D *= 1000, *N*_*d *_= 10, *e *= 0.01 and *μ *= 0.0004. Black bars correspond to *R*_0 _= 1.5 and grey bars to *R*_0 _= 15.

In Figure 5 we show an example of the mutation frequency spectrum in the scale free network for two values of transmission with a large sample size. Clearly we see that when *R*_0 _is small the proportion of singletons in the samples is much higher than when *R*_0 _is large. For *R*_0 _= 15 the spectrum is similar to the one expected under the standard neutral model. These results imply that it is very dificult to reject an equilibrium neutral model with constant population size when using Tajima's D.

### Infectious agent diversity within hosts and differentiation amongst hosts

**Figure 6:**
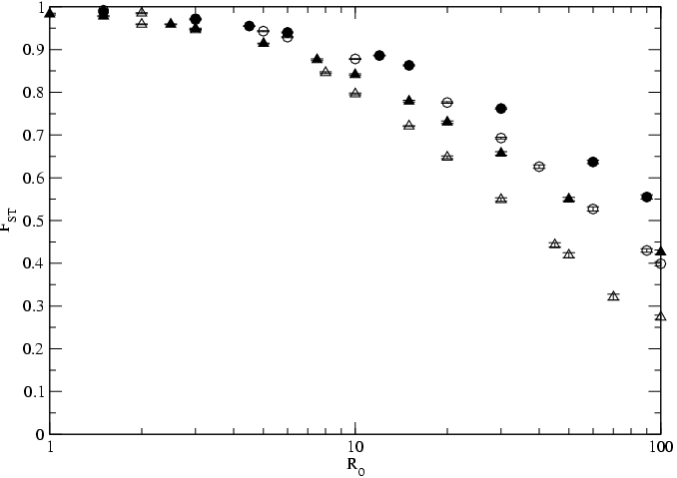
**Level of differentiation**. The level of differentiation among hosts measured as *F*_*ST*_. The empty symbols denote the results for the island model, while the full symbols correspond to scale-free networks. The parameters are *e *= 0.01 (circles) and *e *= 0.02 (triangles), *D *= 1000, *N*_*d *_= 10, *n*_*t *_= 50, *n*_*d *_= 5 and *μ *= 0.0004.

**Figure 7:**
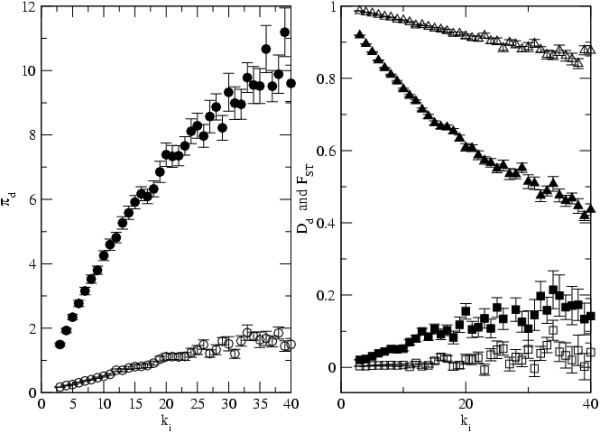
**Within host diversity and differentiation among hosts**. The level of within host diversity, *π*_*d *_(in circles), and differentiation among hosts, *F*_*ST *_(in triangles), as a function of connectivity *k*_*i*_. The squares represent the mean values of Tajima's D within hosts, *D*_*d*_. *R*_0 _= 3 in open symbols and *R*_0 _= 15 in filled symbols, other parameters are *D *= 1000, *N*_*d *_= 10, *e *= 0.01, *n*_*t *_= 50, *n*_*d *_= 5 and *μ *= 0.0004.

In infectious agents with very high mutation rates, as it is the case of RNA viruses [45], one may expect some level of within host diversity to be observed. We have therefore studied the level of diversity in samples taken from each infected host. We also studied the level of genetic differentiation between hosts measured by *F*_*ST *_[46]. The statistic of genetic differentiation we use measures the difference between the level of infectious agent diversity within an infected host and that of the entire infectious agent metapopulation. It is known that all of these statistics are important for the understanding of the relative importance of the processes governing metapopulation dynamics [13] and therefore they can be important in understanding their epidemiology. Figure 6 shows the results for the levels of genetic differentiation, as measured by *F*_*ST*_, for different values of *R*_0_. As can be seen from this figure, for infectious agents with low transmission, the levels of host differentiation are very high. In this case and with the value of the mutation rate considered, the levels of within host genetic variability can be very low. For example in the case of the island model with *R*_0 _= 2.5, the observed mean level of intrahost infectious agent diversity was 0.46, which is only 0.04 of the level observed in the whole metapopulation. In certain RNA viruses, such as the Dengue virus, the levels of intrahost genetic diversity that have been observed are about 0.03 of that between hosts [47]. In infectious agents with high transmission rates the levels of differentiation are much smaller and are accompanied by higher levels of within host diversity. In the case of the island model every host has similar levels of diversity. And so, as the infectious agent transmission rate increases, so does the level of within host diversity. But in scale free networks, host connectivity affects infectious agent diversity within that host and the levels of differentiation between hosts. We have considered the case of two infectious agent with different transmission coeficients and have looked at the relation between host connectivity and within host diversity. Figure 7 shows that well connected hosts have much higher levels of *π*_*d *_and much lower levels of *F*_*ST*_. In this instance, *F*_*ST *_reflects the average divergence between demes with connectivity *k*_*i *_and all other demes. From the figure we see that well connected hosts also show significantly larger mean values of the Tajima's D statistic, for intermediate values of *R*_0_.

## Conclusion

One of the main goals in infectious disease research is to understand how infectious agent variation, host immunity, transmission dynamics and epidemic dynamics determine patterns of infectious agent evolution. Information about evolutionary and epidemiological processes can be extracted from studying infectious agent genetic diversity. In particular it can help us to understand the origin of disease and the selective pressures that act on certain infectious agent genes. The link between infectious agent dynamics and genetic diversity at within and between host level is a very important problem. The means towards its solution requires the integration of population genetics and epidemiology. This has recently been recognized as a major step for understanding infectious agent evolution [5].

Here we have studied levels and patterns of infectious agent diversity under one of the simplest classical epidemiological models: the SIS model. In this model, hosts that are susceptible can become infected at a given rate, and hosts that are infected can become susceptible by clearance of the infectious agent. We have found that, under this model and in the conditions studied, for low clearance rates and low intrahost effective population size, levels of genetic variability in samples from the whole infectious agent population are maximal for intermediate levels of transmission. This pattern of DNA sequence diversity was found to be independent of the type of host contact structure.

Although we have not performed simulations with values of *N*_*d *_close to those that have been estimated for some infectious agent (*N*_*d *_≃ 1000 estimated for HIV-1 [48]) due to the high computational cost, from the simulations we have done we have checked that when the rate at which the immune system clears the infectious agent (*e*) is higher than the rate of drift (1/*Nd*) within the host, levels of infectious agent diversity in the whole metapopulation monotonically increase with *R*_0_.

In highly transmitted infectious agents, levels of diversity are weakly dependent on the type of host contact structure. However for infectious agents with low values of *R*_0_, levels of diversity do depend on the host contact structure: when interactions between hosts are such that every host is in contact with every other, levels of diversity are higher than when the host contact structure is such that a few hosts have a disproportionate number of contacts, whereas the majority has a small number of contacts. In this latter case levels of infectious agent diversity are expected to be low. Furthermore, in this latter case the frequency spectrum of neutral mutations can be distorted, in relation to that expected for the standard neutral model [39]. This feature is captured by negative values of the Tajima's D statistics. The observation of positive values of *D*_*t *_in infections agent genes suggests that strong diversifying selection could be occurring, since even when we account for the complex contact structure in which infectious agents evolve, under a neutral model one would expect to observe values of *D*_*t *_close to 0 or negative.

The results presented here can also be used to make some predictions about future adaptation in infectious agents. If we assume that new adaptive mutations in infectious agents arise from standing neutral variation [49,50], Figures 2, 3 and 4 imply that for infectious agents with low intrahost effective population size, those with intermediate *R*_0 _will be likely to adapt more rapidly than those with larger *R*_0_. For infectious agents in which these conditions are met, an important implication regarding public health measures can be drawn: if control programs with the aim of lowering transmission do not reduce *R*_0 _to very low values, but instead only lead to small reductions in *R*_0_, then this may imply an increased chance of the infectious agent escaping the immune system.

**Figure 8:**
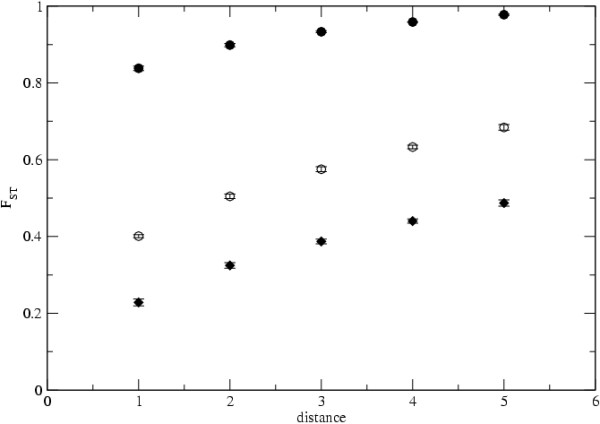
**Level of differentiation between hosts**. The level of differentiation between pairs of hosts, *F*_*ST*_, as a function of their topological distance (which is estimated as the minimum number of links which separates two distinct demes). A scale-free network, with *γ *= 3, is considered with *D *= 500, *N*_*d *_= 10, *μ *= 0.0008, *e *= 0.01. *R*_0 _= 3 in filled circle symbols, *R*_0 _= 30 in empty circle symbols and *R*_0 _= 60 in diamonds.

One feature of several natural populations, including infectious agent populations is the occurrence of correlations between genetic and geographical distance [14,51]. In the island model of population structure that pattern does not arise, whereas in the stepping stone model it is evident. We have explored the relation between genetic and geographical distance in the scale free contact network, which is likely to be closer to the relevant contact structure for infectious agent evolution. Although in our models we have not considered geography explicitly, we have assumed that it can be related to the shortest path length between nodes in the network. Figure 8 shows a clear correlation between these distances. One can intuitively suspect that natural selection can cause infectious agents to adapt to local conditions and that local adaptation can lead to spatial genetic structuring. But before one jumps to the conclusion that natural selection is playing a role in spatially structuring diversity one has to rule out the simpler explanation of neutral evolution in a complex host contact network. Hopefully, the careful consideration of all diversity measures and the use of several test statistics will help us to find the molecular signature of adaptation in infectious agent gene sequences.

## Methods

### General model description

We consider the evolution of a haploid non-recombining population subdivided into small subpopulations-demes. There are *D *demes, each corresponding to a node in the network comprising all the population. Each deme has a maximum size of *N*_*d *_individuals. The total maximum number of individuals in the metapopulation is *N*_*t *_= *DN*_*d*_. Each deme can go extinct with probability *e *and be recolonized through migration of individuals from other demes to which the deme is connected to (see below). Note that in our model recolonization occurs through migration (which is different from other metapopulation models [14]). In order to model migration we do the following. Each deme *i *of a given network is connected to *k*_*i *_other demes according to the specific type of contact network considered. Each edge of the network connects two demes that exchange migrants at a mean rate *m*. We produce a new generation of individuals by taking the following steps: we draw the number of migrants going out from each deme from a Poisson distribution with mean *N*_*d*_*mk*_*i*_, if the deme is not empty. The individuals that migrate are sampled at random, without replacement, from the original deme and added to the recipient demes. The assumption of sampling without replacement, is not restrictive, since we obtain the same results in simulations where sampling with replacement is considered. The relevant parameter of the SIS model is the basic reproductive number *R*_0_, which corresponds to *R*_0 _= *N*_*d*_*mk*/*e *in our model. So in our simulations we changed the value of *R*_0 _by changing the migration rate *m *while keeping constant all other quantities. After migration, reproduction and mutation occurs. *N*_*d *_individuals are chosen at random to form the new population of each deme. Each individual is subject to new mutations following a Poisson distribution with mean *μ*. We assume the infinite sites mutational model where every new mutation occurs at a new site. At the start of each simulation run, all demes have *N*_*d *_individuals, which are mutation-free and are represented by an infinitely large sequence. We then let the simulation run for an initial period, *T*_*eq*_, to allow the metapopulation to reach an epidemiological and genetic equilibrium. The time to reach equilibrium depends on the set of parameters of the simulation. Since all the measurements are obtained after equilibrium, the results do not depend on the initial condition. Every *T *= 5000 generations, after the initial *T*_*e*_*q *generations, we take a sample of size *n*_*t *_= 50 from the entire population, and samples from within each deme of size *n*_*d *_= 5, unless stated otherwise. We then calculate the average number of pairwise differences for the entire population:

πt=∑i<jπijnt(nt−1)/2
 MathType@MTEF@5@5@+=feaafiart1ev1aaatCvAUfKttLearuWrP9MDH5MBPbIqV92AaeXatLxBI9gBaebbnrfifHhDYfgasaacH8akY=wiFfYdH8Gipec8Eeeu0xXdbba9frFj0=OqFfea0dXdd9vqai=hGuQ8kuc9pgc9s8qqaq=dirpe0xb9q8qiLsFr0=vr0=vr0dc8meaabaqaciaacaGaaeqabaqabeGadaaakeaaiiGacqWFapaCdaWgaaWcbaGaemiDaqhabeaakiabg2da9maalaaabaWaaabeaeaacqWFapaCdaWgaaWcbaGaemyAaKMaemOAaOgabeaaaeaacqWGPbqAcqGH8aapcqWGQbGAaeqaniabggHiLdaakeaacqWGUbGBdaWgaaWcbaGaemiDaqhabeaakiabcIcaOiabd6gaUnaaBaaaleaacqWG0baDaeqaaOGaeyOeI0IaeGymaeJaeiykaKIaei4la8IaeGOmaidaaaaa@46E6@

where *π*_*ij *_is the number of differences between two sampled sequences, and also for each deme (*π*_*d*_).

We also calculate the number of segregating sites in each sample (*S*_*t *_and *S*_*d*_) and the test statistic Tajima's D [42] which for samples of the entire population is given by:

Dt=πt−St/antbnt
 MathType@MTEF@5@5@+=feaafiart1ev1aaatCvAUfKttLearuWrP9MDH5MBPbIqV92AaeXatLxBI9gBaebbnrfifHhDYfgasaacH8akY=wiFfYdH8Gipec8Eeeu0xXdbba9frFj0=OqFfea0dXdd9vqai=hGuQ8kuc9pgc9s8qqaq=dirpe0xb9q8qiLsFr0=vr0=vr0dc8meaabaqaciaacaGaaeqabaqabeGadaaakeaacqWGebardaWgaaWcbaGaemiDaqhabeaakiabg2da9maalaaabaacciGae8hWda3aaSbaaSqaaiabdsha0bqabaGccqGHsislcqWGtbWudaWgaaWcbaGaemiDaqhabeaakiabc+caViabdggaHnaaBaaaleaacqWGUbGBdaWgaaadbaGaemiDaqhabeaaaSqabaaakeaacqWGIbGydaWgaaWcbaGaemOBa42aaSbaaWqaaiabdsha0bqabaaaleqaaaaaaaa@41A4@

where an=∑i=1,n1i
 MathType@MTEF@5@5@+=feaafiart1ev1aaatCvAUfKttLearuWrP9MDH5MBPbIqV92AaeXatLxBI9gBaebbnrfifHhDYfgasaacH8akY=wiFfYdH8Gipec8Eeeu0xXdbba9frFj0=OqFfea0dXdd9vqai=hGuQ8kuc9pgc9s8qqaq=dirpe0xb9q8qiLsFr0=vr0=vr0dc8meaabaqaciaacaGaaeqabaqabeGadaaakeaacqWGHbqydaWgaaWcbaGaemOBa4gabeaakiabg2da9maaqababaWaaSaaaeaacqaIXaqmaeaacqWGPbqAaaaaleaacqWGPbqAcqGH9aqpcqaIXaqmcqGGSaalcqWGUbGBaeqaniabggHiLdaaaa@3A6C@, *b*_*n *_= *e*_1_*S *+ *e*_2_*S*(*S *- 1) and *e*_1 _*e*_2 _as defined by Tajima [42].

One other quantity of interest that we have studied is *F*_*ST*_, a measure of genetic differentiation amongst demes. This measure is defined as [46]:

FST=πt−πdπt
 MathType@MTEF@5@5@+=feaafiart1ev1aaatCvAUfKttLearuWrP9MDH5MBPbIqV92AaeXatLxBI9gBaebbnrfifHhDYfgasaacH8akY=wiFfYdH8Gipec8Eeeu0xXdbba9frFj0=OqFfea0dXdd9vqai=hGuQ8kuc9pgc9s8qqaq=dirpe0xb9q8qiLsFr0=vr0=vr0dc8meaabaqaciaacaGaaeqabaqabeGadaaakeaacqWGgbGrdaWgaaWcbaGaem4uamLaemivaqfabeaakiabg2da9maalaaabaacciGae8hWda3aaSbaaSqaaiabdsha0bqabaGccqGHsislcqWFapaCdaWgaaWcbaGaemizaqgabeaaaOqaaiab=b8aWnaaBaaaleaacqWG0baDaeqaaaaaaaa@3C59@

A well studied topology in the population genetics literature is the island model, introduced by Wright, which corresponds to a fully connected network where every deme is connected to the others, so *k*_*i *_= *D *- 1. A commonly studied topology in epidemiology is the scale-free network, where the distribution of connectivities obeys a power-law: P(ki)∝ki−γ
 MathType@MTEF@5@5@+=feaafiart1ev1aaatCvAUfKttLearuWrP9MDH5MBPbIqV92AaeXatLxBI9gBaebbnrfifHhDYfgasaacH8akY=wiFfYdH8Gipec8Eeeu0xXdbba9frFj0=OqFfea0dXdd9vqai=hGuQ8kuc9pgc9s8qqaq=dirpe0xb9q8qiLsFr0=vr0=vr0dc8meaabaqaciaacaGaaeqabaqabeGadaaakeaacqWGqbaucqGGOaakcqWGRbWAdaWgaaWcbaGaemyAaKgabeaakiabcMcaPiabg2Hi1kabdUgaRnaaDaaaleaacqWGPbqAaeaacqGHsisliiGacqWFZoWzaaaaaa@3979@. In real systems the exponent *γ *is in the range between 2 and 3. Nodes of low connectivity are predominant in the network, whereas well-connected nodes are rare. One of the mechanisms that can lead to the occurrence of a network with a power-law degree distribution is growth with preferential attachment, where nodes newly introduced to the network are preferentially attached to those nodes which are already well connected. We use the standard algorithm by Albert and Barabasi to build up the scale-free networks [21], and so we generate networks with exponent *γ *= 3. Scale free networks, that are extremely heterogeneous, may be appropriate descriptions for studying sexually transmitted diseases [18]. Our results for scale-free networks were compared to the island model. For every network and every parameter set we have run 30 independent simulations.

## Authors' contributions

The authors contributed equally to this work.
